# Dose optimization of remimazolam as an adjunct to propofol for gastroscopy sedation: a randomized double-blind trial

**DOI:** 10.3389/fphar.2026.1782559

**Published:** 2026-04-29

**Authors:** Yijie Zhang, Shanshan Xia, Hai Wang, Hanbing Shen, Lian Fang, Yating Chen, Yuanyuan Pan, Nana Bao, Lili Yang, Yingchao Ye, Junlu Wang, Yunchang Mo

**Affiliations:** 1 Department of Anesthesiology, The Jinhua Affiliated Hospital of Wenzhou Medical University, Jinhua, China; 2 Department of Anesthesiology, The First Affiliated Hospital of Wenzhou Medical University, Wenzhou, China; 3 Department of Anesthesiology, Wenzhou People's Hospital, Wenzhou, China; 4 Hepatobiliary Pancreatic Gastrointestinal Surgery, The Jinhua Affiliated Hospital of Wenzhou Medical University, Jinhua, China

**Keywords:** dose optimization, EC50, gastroscopy, procedural sedation, propofol, randomized controlled trial, remimazolam

## Abstract

**Introduction:**

Propofol-based sedation for gastroscopy is effective but may cause dose-dependent cardiorespiratory depression; therefore, optimizing adjunct strategies to reduce propofol exposure is clinically important.

**Methods:**

We conducted a prospective, single-center, randomized, double-blind, dose-response trial to evaluate the propofol-sparing effect of remimazolam during procedural sedation for gastroscopy. A total of 150 ASA I-II adults aged 18–59 years were randomly assigned to receive remimazolam 0, 0.05, 0.1, 0.15, or 0.2 mg·kg^−1^, and propofol was titrated to achieve adequate sedation. The primary outcomes were the propofol effect-site EC50 and propofol requirement (mg·kg^−1^·min^−1^). Secondary outcomes included recovery profiles, post-anesthesia care unit (PACU) duration, and sedation-related adverse events.

**Results:**

Remimazolam reduced the propofol EC50 in a dose-dependent manner, from 3.05 (95% CI, 2.88–3.21) μg·mL^−1^ at 0.05 mg·kg^−1^ to 1.47 (95% CI, 1.30–1.64) μg·mL^−1^ at 0.2 mg·kg^−1^, and decreased propofol requirement from 0.587 ± 0.193 mg·kg^−1^·min^−1^ in the control group to 0.414 ± 0.121 mg·kg^−1^·min^−1^ at 0.1 mg·kg^−1^ and 0.298 ± 0.088 mg·kg^−1^·min^−1^ at 0.2 mg·kg^−1^. Procedure duration and PACU duration did not differ significantly among groups, and the incidence of sedation-related adverse events was comparable.

**Discussion:**

Adjunctive remimazolam provided a dose-dependent propofol-sparing effect during gastroscopy sedation. Among the tested doses, 0.1 mg·kg^−1^ appeared to offer a favorable balance between reduced propofol requirement and recovery profile, supporting its clinical feasibility under the conditions studied.

**Clinical Trial Registration:**

Chinese Clinical Trial Registry, identifier ChiCTR2400094449.

## 1 Introduction

Gastrointestinal endoscopy is a common outpatient procedure, and sedated gastroscopy is routinely used to enhance patient comfort and diagnostic yield (Agostoni et al., 2011; Behrens et al., 2019; Goudra et al., 2020). In China, the most widely used sedation strategy for gastroscopy is deep sedation without tracheal intubation, most commonly a propofol-based regimen combined with opioids (Committee et al., 2018; Zhou et al., 2021). However, both propofol and opioids exert dose-dependent depressive effects on respiratory and cardiovascular function, and their combined use may increase the risk of hypotension and respiratory depression, particularly in elderly patients and those with underlying comorbidities (Schug et al., 1992; Wang et al., 2023).

Remimazolam is a novel, ultra-short-acting benzodiazepine and a γ-aminobutyric acid type A receptor agonist with rapid onset, short context-sensitive half-time, minimal respiratory and circulatory depression, absence of injection pain, and the availability of a specific antagonist (flumazenil) (Chen et al., 2021; Wang et al., 2022). Although remimazolam alone provides adequate sedation for endoscopy, it is associated with a higher incidence of patient movement and lower endoscopist satisfaction scores compared with propofol-based regimens (Borkett et al., 2015; Zhu et al., 2024). Recent studies have further demonstrated that combining remimazolam with propofol reduces adverse events compared with propofol alone and improves sedation quality and endoscopist satisfaction compared with remimazolam alone (Barbosa et al., 2024; Wei et al., 2023).

However, the dose–response characteristics of remimazolam when combined with propofol for gastroscopy have not been systematically established. In particular, the effects of different remimazolam doses on the effect-site concentration of propofol (Ceprop), propofol-sparing effects, and recovery profiles remain unclear. Therefore, this randomized, double-blind trial was designed to evaluate the dose–response relationship between remimazolam and propofol during gastroscopy and to determine the effect of different doses of remimazolam on the propofol EC50 required to achieve adequate sedation. Secondary objectives were to assess the effects of remimazolam supplementation on total propofol consumption, emergence and recovery times, and safety.

## 2 Methods

### 2.1 Study design and subjects

This prospective, single-center, randomized, double-blind, dose–response trial was conducted in the Department of Anesthesiology, The First Affiliated Hospital of Wenzhou Medical University, Wenzhou, China, from 1 January 2025 to 28 February 2025. The study was reported in accordance with the CONSORT 2010 statement and the CONSORT extension for dose-finding studies. The study protocol was approved by the Ethics Committee in Clinical Research (ECCR) of the First Affiliated Hospital of Wenzhou Medical University (Ref: KY 2024-245, approved on 20 December 2024) and was prospectively registered at the Chinese Clinical Trial Registry (ChiCTR2400094449, registered on 23 December 2024). Written informed consent was obtained from all participants before enrollment.

We enrolled 150 ASA I–II adults aged 18–59 years with BMI 17.5–29 kg·m^-2^. Additional inclusion criteria were elective gastroscopy and the ability to understand the study procedures and comply with the protocol. Key exclusion criteria included acute respiratory infection within 1 week, cardiovascular disease with angina and/or dyspnoea, psychiatric disorders, severe hepatic or renal dysfunction, history of alcohol or psychoactive drug abuse within the past 2 years, use of opioids, non-steroidal anti-inflammatory drugs, or paracetamol within 24 h before the procedure, obstructive sleep apnea syndrome, known allergy to propofol or remimazolam, difficult airway (Mallampati class ≥ III, inter-incisor distance <3 cm, or cervical immobility), and active gastrointestinal bleeding.

The total sample size of 150 participants (30 per group) was determined based on previous dose-finding studies using the Dixon up-and-down method and on a similar clinical EC_50_ study of remimazolam–propofol combination during hysteroscopic procedures (Zhou et al., 2024), as well as established methodological recommendations for dose-finding designs (Dixon, 1991; Pace and Stylianou, 2007; Paul and Fisher, 2001).

### 2.2 Randomization and blinding

**FIGURE 1:**
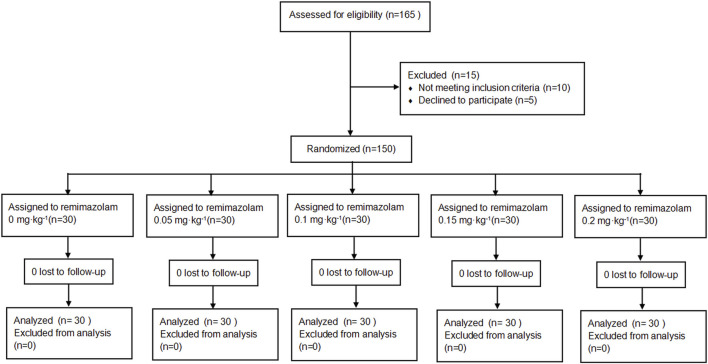
CONSORT flow diagram. A total of 165 patients were assessed for eligibility, of whom 15 were excluded (10 not meeting the inclusion criteria and 5 declining participation). Overall, 150 patients were randomized to receive remimazolam 0, 0.05, 0.1, 0.15, or 0.2 mg·kg^-1^ (n = 30 per group). No patients were lost to follow-up, and all randomized patients were included in the final analysis.

A total of 150 eligible patients were randomly assigned to five groups (0, 0.05, 0.1, 0.15, or 0.2 mg·kg^-1^ remimazolam; n = 30 each) using a computer-generated random sequence with fixed block sizes of five. Allocation concealment was ensured by pharmacy-prepared, sequentially numbered, sealed, opaque envelopes that were stored in a locked cabinet accessible only to an unblinded technician. Immediately before each procedure, the technician opened the envelope and prepared the study drug according to group assignment. Remimazolam was supplied as a 36 mg vial, reconstituted and further diluted with 0.9% saline to a final concentration of 1 mg·mL^-1^. The technician then loaded 36 mL of the 1 mg·mL^-1^ remimazolam solution into an identical 50 mL syringe. For the 0 mg·kg^-1^ group, 36 mL of 0.9% saline was prepared in an identical 50 mL syringe using the same procedure. All syringes were labeled only with a unique randomization number and were visually indistinguishable. The attending anesthesiologists, patients, and investigators for data collection and assessment were blinded to group allocation. The unblinded technician had no further involvement in patient care, data collection, or outcome assessment. Patient enrollment and randomization were detailed in the CONSORT flow diagram (Figure 1).

### 2.3 Study protocol

All patients fasted for 6–8 h and had no clear liquids for 2 h before the procedure. Standard preoperative assessments included complete blood count, coagulation profile, electrocardiography, and anesthetic evaluation. No premedication was administered. Standard monitoring consisted of electrocardiography (ECG), noninvasive blood pressure (NIBP), heart rate (HR), pulse oximetry (SpO_2_), and bispectral index (BIS). Oxygen was administered at 6 L·min^-1^ via face mask. After intravenous access was established, the assigned study drug (remimazolam 0–0.2 mg·kg^-1^; 0 mg·kg^-1^ group received 0.9% saline) was administered via a syringe pump over 60 s. For each participant, the dose was calculated based on body weight and the corresponding delivery volume (mL) was calculated using the prepared concentration (1 mg·mL^-1^). The pump was programmed to deliver the calculated volume over 60 s (infusion rate (mL·h^-1^) = volume (mL) × 60; maximum allowable rate 999 mL·h^-1^). Propofol was administered using a TCI-V target-controlled infusion system (Guangxi Weili Fangzhou Technology Co., Ltd., Guangxi, China) implementing the Marsh pharmacokinetic model for effect-site targeting. All propofol concentrations reported in this study refer to the TCI-predicted target effect-site concentration (Ce) rather than directly measured plasma concentrations. The software version and ke_0_value were not displayed on the device interface and were not user-accessible.

The Dixon up-and-down sequential method was used to determine the propofol EC_50_. The up-and-down sequence was conducted separately within each remimazolam dose group. The first patient in each group received a preset target propofol effect-site concentration (Ceprop) of 3.0 μg·mL^-1^, which produced loss of consciousness (i.e., loss of response to verbal commands and loss of the eyelash reflex) in most patients. Endoscopy was initiated by an experienced endoscopist when the eyelash reflex disappeared, the Modified Observer’s Assessment of Alertness/Sedation (MOAA/S) score was ≤2 (Supplementary Table S1), and BIS decreased to 40–60 (Supplementary Table S2). Sedation success was defined as loss of the eyelash reflex, MOAA/S ≤ 2, BIS 40–60, and no or mild movement or coughing not interfering with the procedure. Sedation failure was defined as marked movement or coughing interfering with endoscope insertion, or BIS >60, during endoscope insertion. In cases of sedation failure, the target Ceprop was increased by 0.5 μg·mL^-1^ every 2.5 min until sedation success was achieved. If BIS decreased below 40, the target Ceprop was reduced by 0.5 μg·mL^-1^. For subsequent patients, the initial Ceprop was adjusted by ± 0.5 μg·mL^-1^ according to the sedation outcome of the preceding patient. During endoscopy, the propofol target concentration was adjusted as necessary to maintain BIS between 40 and 60, and infusion was discontinued at the end of the procedure. All patients were transferred to the post-anesthesia care unit (PACU) after endoscopy.

Data collection included demographic characteristics (age, sex, height, weight, and BMI) and instantaneous Ceprop at loss of consciousness (C1), endoscope insertion (C2), discontinuation of propofol (C3), and eye opening (C4). Hemodynamic variables (NIBP, HR, and SpO_2_) were recorded at four time points: room entry (T1), endoscope insertion (T2), loss of consciousness (T3), and eye opening (T4). Adverse events were defined as follows: hypotension was defined as systolic blood pressure <90 mmHg or <80% of baseline and was treated with intravenous norepinephrine 4 μg, repeated as needed; bradycardia was defined as a heart rate <50 beats·min^-1^ and was treated with intravenous atropine 0.5 mg; desaturation was defined as SpO_2_ < 95% to capture early oxygen desaturation events and was managed by lifting the lower jaw and/or assisted ventilation via a facemask, and if unresolved, by placement of a nasopharyngeal airway or endotracheal intubation. Other adverse events included nausea and vomiting, coughing, injection pain, dizziness, and postoperative pain.

Secondary outcomes included total propofol consumption, propofol requirement (mg·kg^-1^·min^-1^), procedure duration, anesthesia emergence time (from discontinuation of propofol to eye opening on command), PACU duration (from completion of endoscopy to PACU discharge), and pain intensity, which was assessed using the visual analog scale (VAS) (Supplementary Table S3) at 30 min after arrival in the PACU.

### 2.4 Statistical analysis

Studies have shown that 20–40 patients are sufficient to obtain at least six pairs of sequence reversals for calculating EC_50_ using the Dixon up-and-down method (Zhou et al., 2024; Dixon, 1991; Pace and Stylianou, 2007; Paul and Fisher, 2001). In this study, the up-and-down sequence was conducted separately within each remimazolam dose group. Because dose allocation in the Dixon up-and-down design is determined by the preceding participant’s response, the sequential observations are not statistically independent; therefore, the raw sequential concentrations were not subjected to conventional independent-sample group comparisons; between-group differences in EC_50_ were described using relative mean potency and 95% CIs.Between-group comparisons of EC_50_ should therefore be interpreted with caution given the sequential nature of the design. For continuous variables other than the primary EC_50_ endpoint, the Kolmogorov–Smirnov test was used to assess normality. Normally distributed variables were presented as mean ± standard deviation (SD) and were compared using one-way analysis of variance (ANOVA). Non-normally distributed variables were presented as median (interquartile range, IQR) and were compared using the Kruskal–Wallis test. Categorical variables were presented as numbers (percentages) and were analyzed using the χ^2^ test or Fisher’s exact test, as appropriate.

The 95% confidence intervals (CIs) for EC_50_ values were calculated using Choi’s method (Choi, 1990). In this study, EC_50_ refers to the median effective target effect-site concentration (Ce) of propofol predicted by the Marsh model-based TCI system, rather than a directly measured plasma concentration. Estimates of propofol EC_50_ for each group were obtained, and differences among the five groups were quantified by calculating the relative mean potency with 95% CIs, as described previously (Choi, 1990; Choi, 1971). Probit regression analysis was additionally performed as a sensitivity analysis, analyzing tallied effectiveness and ineffectiveness counts for each dose category per group (Pace and Stylianou, 2007). Propofol requirements (mg·kg^-1^·min^-1^) were calculated as total propofol consumption divided by (anesthesia duration × body weight).

All statistical analyses were performed using SPSS software (version 27.0; IBM, Armonk, NY, USA) and GraphPad Prism (version 10.0; GraphPad Software, San Diego, CA, USA). The statistician was blinded to group allocation until completion of data analysis. A two-sided *P* < 0.05 was considered statistically significant. For *post hoc* pairwise comparisons among the five groups, Bonferroni correction was applied (10 pairwise comparisons), and *P* < 0.005 was considered statistically significant.

## 3 Results

### 3.1 Patient characteristics

Between 1 January 2025 and 28 February 2025, 165 patients were screened for elective gastroscopy. Among these, 10 patients did not meet the inclusion criteria and 5 patients declined to participate. A total of 150 patients were randomized into five groups (n = 30 per group) and were included in the final analysis (Figure 1). Baseline demographic and clinical characteristics, including age, body mass index (BMI), height, weight, and sex were comparable among the five groups (all *P* > 0.05; Table 1).

**TABLE 1:** Patient characteristics.

Characteristic	Remimazolam 0 mg·kg^-1^ (n = 30)	Remimazolam 0.05 mg·kg^-1^ (n = 30)	Remimazolam 0.1 mg·kg^-1^ (n = 30)	Remimazolam 0.15 mg·kg^-1^ (n = 30)	Remimazolam 0.2 mg·kg^-1^ (n = 30)	*P* Value
Age (y)	46.83 ± 9.20	42.13 ± 9.85	45.63 ± 10.15	42.00 ± 13.06	41.93 ± 12.51	0.254
BMI (kg·m^-2^)	21.92 ± 2.43	23.35 ± 3.34	22.29 ± 2.96	23.06 ± 2.62	21.81 ± 2.68	0.139
Height (cm)	161.37 ± 6.81	164.67 ± 8.14	164.10 ± 8.63	164.93 ± 8.03	163.83 ± 7.80	0.428
Weight (kg)	57.10 ± 7.19	63.60 ± 11.66	60.23 ± 10.42	62.97 ± 10.09	58.80 ± 9.78	0.062
Male, n (%)	6 (20.0%)	14 (46.7%)	13 (43.3%)	15 (50.0%)	12 (40.0%)	0.139

Data are presented as mean ± SD, or N (%).

Abbreviations: BMI, body mass index; SD, standard deviation.

### 3.2 Propofol EC_50_

**FIGURE 2:**
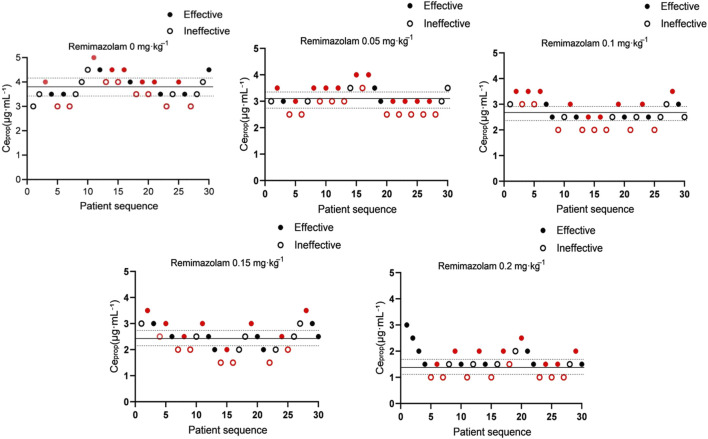
Sequential up-and-down plots of propofol effect-site concentration (Ceprop) by remimazolam dose. Each panel shows the target Ceprop administered to consecutive patients (n = 30) in the corresponding remimazolam group (0, 0.05, 0.1, 0.15, and 0.2 mg·kg^-1^). Filled circles indicate successful sedation, defined as loss of the eyelash reflex, a Modified Observer’s Assessment of Alertness/Sedation (MOAA/S) score ≤2, BIS 40–60, and no or only mild movement or coughing not interfering with endoscopy. Open circles indicate sedation failure, defined as marked movement, coughing, or BIS >60 during endoscope insertion. Red filled circles and red open circles denote success and failure at reversal points, respectively, and were used for EC_50_ estimation. The solid horizontal line indicates the estimated propofol EC_50_ derived from the up-and-down method, and the dashed lines indicate the 95% confidence interval (CI). Ceprop, effect-site propofol concentration; EC_50_, Ceprop associated with a 50% probability of successful sedation.

Patient responses to Ceprop during sedated gastroscopy were shown in Figure 2. The propofol EC50 for achieving adequate sedation, calculated using the up-and-down method, was 3.81 μg·mL^-1^ (95% CI, 3.57–4.05) in the 0 mg·kg^-1^ remimazolam group and was lower in patients receiving remimazolam at 0.05 mg·kg^-1^ [3.05 (95% CI, 2.88–3.21) μg·mL^-1^], 0.1 mg·kg^-1^ [2.69 (95% CI, 2.47–2.92) μg·mL^-1^], 0.15 mg·kg^-1^ [2.38 (95% CI, 2.10–2.67) μg·mL^-1^], and 0.2 mg·kg^-1^ [1.47 (95% CI, 1.30–1.64) μg·mL^-1^]. Compared with the control group, remimazolam at doses of 0.05, 0.1, 0.15, and 0.2 mg·kg^-1^ reduced the EC_50_ of propofol by 19.9% (95% CI, 13.0%–26.3%), 29.4% (95% CI, 21.6%–36.4%), 37.5% (95% CI, 28.5%–45.4%), and 61.4% (95% CI, 56.0%–66.2%), respectively.

**FIGURE 3:**
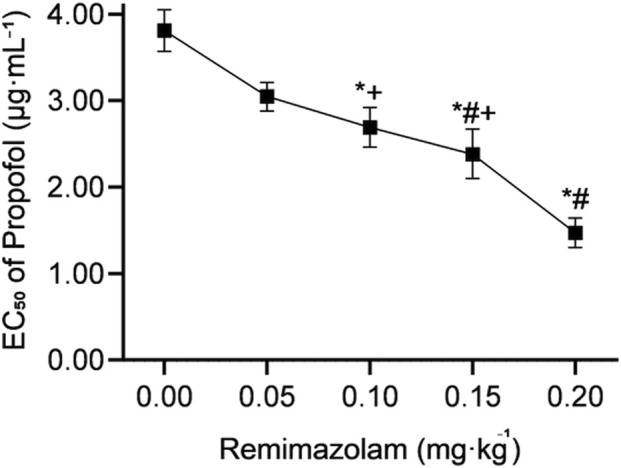
Propofol EC_50_ by remimazolam dose. Propofol EC_50_ (µg·mL^-1^) for achieving adequate sedation is shown for remimazolam 0, 0.05, 0.1, 0.15, and 0.2 mg·kg^-1^. Data are presented as EC_50_ with 95% confidence intervals (CI). ^*^ *P* < 0.005, compared with remimazolam 0 mg·kg^-1^ after Bonferroni correction. ^#^ *P* < 0.005, compared with remimazolam 0.05 mg·kg^-1^ after Bonferroni correction. ^+^ *P* < 0.005, compared with remimazolam 0.2 mg·kg^-1^ after Bonferroni correction.

Numerically, the estimated EC_50_ was lower in the 0.15 mg·kg^-1^ group than in the 0.05 mg·kg^-1^ group and lower in the 0.2 mg·kg^-1^ group than in the 0, 0.05, 0.1 and 0.15 mg·kg^-1^ groups. The differences were less apparent between the 0.05 and 0.1 mg·kg^-1^ groups or between the 0.1 and 0.15 mg·kg^-1^ groups (Figure 3). Probit regression analysis yielded consistent results, supporting that the EC_50_ of propofol was lower in patients receiving remimazolam at all four doses than in those receiving 0 mg·kg^-1^ remimazolam (Table 2). Dose-response curves derived from probit regression analysis are presented in Supplementary Figure S1. Under the parallel-slope probit model, the estimated Hill Slopes of the dose-response curves were the same in all groups (1.771 [95% CI, 1.210–2.332]).

**TABLE 2:** EC_50_ of propofol (µg·mL^-1^) for achieving effective sedation during gastroscopy.

Outcome	Remimazolam 0 mg·kg^-1^	Remimazolam 0.05 mg·kg^-1^	Remimazolam 0.1 mg·kg^-1^	Remimazolam 0.15 mg·kg^-1^	Remimazolam 0.2 mg·kg^-1^	Effect size (eta-squared)
EC_50_ ^a^	3.81 (3.57–4.05)^+^	3.05 (2.88–3.21)^*+^	2.69 (2.47–2.92)^*+^	2.38 (2.10–2.67)^*#+^	1.47 (1.30–1.64)^*#^	0.767
Decrease from baseline	Ref	19.9% (13.0–26.3)	29.4% (21.6–36.4)	37.5% (28.5–45.4)	61.4% (56.0–66.2)	-
EC_50_ ^b^	3.84 (3.55–4.15)	3.14 (2.86–3.42)	2.68 (2.40–2.97)	2.39 (2.10–2.68)	1.47 (1.19–1.75)	-

Data are presented as mean (95% CI); n = 30 per group.

Abbreviations: CI, confidence interval; EC_50_, effect-site propofol concentration (Ceprop) required to achieve successful sedation in 50% of patients.

^a^ Dixon’s up-and-down method.

^b^ probit regression analysis.

* *P* < 0.005 compared with remimazolam 0 mg·kg-1, after Bonferroni correction.

# *P* < 0.005 compared with remimazolam 0.05 mg·kg-1, after Bonferroni correction.

+*P* < 0.005 compared with remimazolam 0.2 mg·kg^-1,^ after Bonferroni correction.

At the four time points—loss of consciousness, endoscope insertion, discontinuation of propofol, and eye opening on command—the instantaneous Ceprop was significantly lower in patients receiving remimazolam at 0.05, 0.1, 0.15, and 0.2 mg·kg^-1^ than in those receiving 0 mg·kg^-1^ (all *P* < 0.005). Compared with the 0.05 mg·kg^-1^ group, Ceprop was further reduced in the 0.15 and 0.2 mg·kg^-1^ groups (both *P* < 0.005). In addition, the 0.2 mg·kg^-1^ group showed a lower Ceprop than the 0.1 mg·kg^-1^ group (*P* < 0.005). At the time of eye opening on command, Ceprop was lower in the 0.15 and 0.2 mg·kg^-1^ groups than in the 0 and 0.05 mg·kg^-1^ groups (both *P* < 0.005) (Table 3).

**TABLE 3:** Ceprop during sedated gastroscopy.

Timepoint	Remimazolam 0 mg·kg^-1^	Remimazolam 0.05 mg·kg^-1^	Remimazolam 0.1 mg·kg^-1^	Remimazolam 0.15 mg·kg^-1^	Remimazolam 0.2 mg·kg^-1^
C1(Ce at loss of consciousness)	1.67 (1.51–1.84)	1.17 (1.02–1.33)^a^	0.75 (0.62–0.88)^a^	0.53 (0.44–0.62)^a^ ^,^ ^b^	0.44 (0.37–0.52)^a^ ^,^ ^b^ ^,^ ^c^
C2(Ce at endoscope insertion)	3.78 (3.58–3.98)	3.08 (2.92–3.25)^a^	2.68 (2.50–2.86)^a^	2.48 (2.28–2.69)^a^ ^,^ ^b^	1.61 (1.43–1.79)^a^ ^,^ ^b^ ^,^ ^c^
C3(Ce at discontinuation of propofol)	4.19 (4.00–4.38)	3.42 (3.22–3.62)^a^	3.00 (2.85–3.16)^a^	2.84 (2.64–3.04)^a^ ^,^ ^b^	1.98 (1.79–2.17)^a^ ^,^ ^b^ ^,^ ^c^
C4(Ce at eye opening)	2.55 (2.30–2.80)	1.58 (1.43–1.74)^a^	1.38 (1.19–1.56)^a^	1.09 (0.97–1.21)^a^ ^,^ ^b^	0.80 (0.69–0.92)^a^ ^,^ ^b^ ^,^ ^c^

Data are presented as mean (95% CI).

Abbreviations: Ceprop, effect-site propofol concentration; CI, confidence interval.

^a^ *P* < 0.005 compared with remimazolam 0 mg·kg-1, after Bonferroni correction.

^b^ *P* < 0.005 compared with remimazolam 0.05 mg·kg-1, after Bonferroni correction.

^c^ *P* < 0.005 compared with remimazolam 0.1 mg·kg-1, after Bonferroni correction.

### 3.3 Periprocedural outcomes and adverse events

**FIGURE 4:**
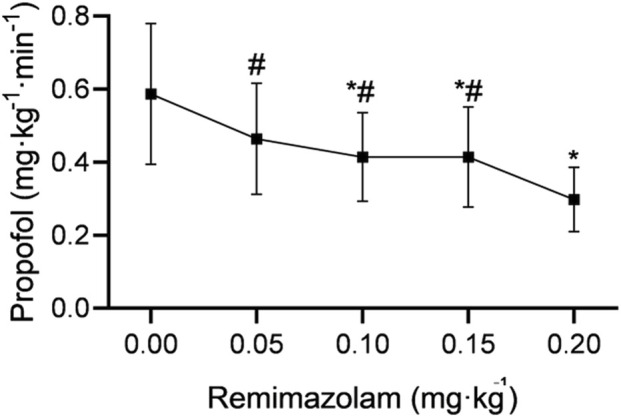
Propofol requirement by remimazolam dose. Propofol requirement (mg·kg^-1^·min^-1^), calculated as total propofol consumption divided by anesthesia duration and body weight, is shown for remimazolam 0, 0.05, 0.1, 0.15, and 0.2 mg·kg^-1^. Data are presented as mean ± SD. ^*^ *P* < 0.005, compared with remimazolam 0 mg·kg^-1^ after Bonferroni correction. ^#^ *P* < 0.005, compared with remimazolam 0.2 mg·kg^-1^ after Bonferroni correction.

Propofol requirement (total propofol dose/[anesthesia duration × body weight]) was significantly lower in patients receiving remimazolam at 0.1 mg·kg^-1^ (0.414 ± 0.121 mg·kg^-1^·min^-1^), 0.15 mg·kg^-1^ (0.414 ± 0.137 mg·kg^-1^·min^-1^), and 0.2 mg·kg^-1^ (0.298 ± 0.088 mg·kg^-1^·min^-1^) than in those receiving 0 mg·kg^-1^ (0.587 ± 0.193 mg·kg^-1^·min^-1^) (all *P* < 0.005). Propofol requirement was also significantly lower in the 0.2 mg·kg^-1^ group than in the 0.05 mg·kg^-1^ (0.464 ± 0.152 mg·kg^-1^·min^-1^), 0.1 mg·kg^-1^, and 0.15 mg·kg^-1^ groups (all *P* < 0.005). No significant differences in propofol requirement were observed between the 0 and 0.05 mg·kg^-1^ groups, between the 0.05 and 0.1 mg·kg^-1^ groups, between the 0.05 and 0.15 mg·kg^-1^ groups, or between the 0.1 and 0.15 mg·kg^-1^ groups (all *P* > 0.05; Table 4; Figure 4).

**TABLE 4:** Postoperative data.

Outcome	Remimazolam 0 mg·kg^-1^	Remimazolam 0.05 mg·kg^-1^	Remimazolam 0.1 mg·kg^-1^	Remimazolam 0.15 mg·kg^-1^	Remimazolam 0.2 mg·kg^-1^
Propofol requirement (mg·kg^-1^·min^-1^)	0.587 ± 0.193	0.464 ± 0.152^#^	0.414 ± 0.121^*#^	0.414 ± 0.137^*#^	0.298 ± 0.088^*^
Time to eye opening (min)	7.9 ± 2.4	8.7 ± 2.9	9.4 ± 2.8	11.0 ± 3.4^*^	10.6 ± 3.1^*^
PACU duration (min)	20.3 ± 4.4	19.9 ± 4.7	21.1 ± 5.0	22.5 ± 5.1	22.0 ± 5.2

Data are presented as mean ± SD, or n (%), unless otherwise indicated.

Abbreviation: PACU, postanesthesia care unit.

* *P* < 0.005 compared with remimazolam 0 mg·kg-1, after Bonferroni correction.

# *P* < 0.005 compared with remimazolam 0.2 mg·kg-1, after Bonferroni correction.

**FIGURE 5:**
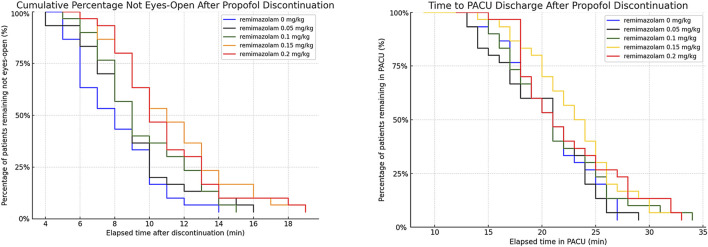
Kaplan–Meier curves for time to PACU discharge. The curves show the percentage of patients remaining in the post-anesthesia care unit (PACU) over time across the five remimazolam groups. Time zero was defined as propofol discontinuation. Overall, PACU discharge profiles were similar among groups. Kaplan–Meier curves for time to eye opening after propofol discontinuation. The curves show the percentage of patients remaining unconscious over time after discontinuation of propofol, with eye opening on command defined as the event. Time zero was defined as propofol discontinuation. The median time to eye opening increased with remimazolam dose, and the 0.15 and 0.2 mg·kg^-1^ group exhibited a slower awakening profile than the 0 mg·kg^-1^ group.

Procedure duration and PACU duration did not differ significantly among the five groups (all *P* > 0.05; Table 4). Time to eye opening differed significantly among groups: patients receiving remimazolam at 0.15 mg·kg^-1^ (11.0 ± 3.4 min) and 0.2 mg·kg^-1^ (10.6 ± 3.1 min) had longer time to eye opening than those receiving 0 mg·kg^-1^ (7.9 ± 2.4 min) (both *P* < 0.005). No significant differences were observed among the 0 mg·kg^-1^ (7.9 ± 2.4 min), 0.05 mg·kg^-1^ (8.7 ± 2.9 min), and 0.1 mg·kg^-1^ (9.4 ± 2.8 min) groups (all *P* > 0.05; Table 4; Figure 5).

**FIGURE 6:**
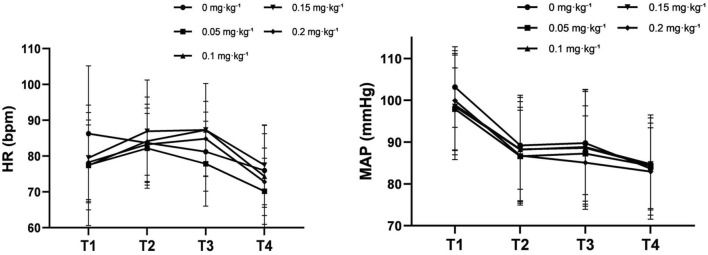
Hemodynamic time course during sedated gastroscopy across remimazolam dose groups. Time-course of heart rate (HR, A) and mean arterial pressure (MAP, B) during the peri-procedural period. Data are presented as mean ± SD for the five remimazolam dose groups at four predefined time points: T1 (room entry), T2 (endoscope insertion), T3 (loss of consciousness), and T4 (eye opening). No significant between-group differences were observed at any time point (all *P* > 0.05).

Hemodynamic variables are summarized in Figure 6. Within-group comparisons showed that mean arterial pressure (MAP) was higher at T1 than at T2, T3, and T4 (all *P* < 0.005), and higher at T2 and T3 than at T4 (all *P* < 0.005). Heart rate (HR) was lower at T1 than at T2 and T3 and higher at T1, T2, and T3 than at T4 (all *P* < 0.005). Between-group comparisons revealed no significant differences in MAP and HR at any time point (all *P* > 0.05; Supplementary Table S4).

No patient experienced bradycardia, nausea and vomiting, or intraoperative awareness. The incidences of hypotension, desaturation, body movement, coughing, dizziness, sore throat and hiccups did not differ significantly among the five groups (all *P* > 0.05). The incidence of injection pain was significantly lower in patients receiving remimazolam at 0.05, 0.1, 0.15, and 0.2 mg·kg^-1^ than in those receiving 0 mg·kg^-1^ (all *P* < 0.005; Table 5).

**TABLE 5:** Comparison of adverse events among the five groups.

Adverse events	Group					*χ* ^*2*^	*P*
	Remimazolam 0 mg·kg^-1^	Remimazolam 0.05 mg·kg^-1^	Remimazolam 0.1 mg·kg^-1^	Remimazolam 0.15 mg·kg^-1^	Remimazolam 0.2 mg·kg^-1^		
Injection pain	14 (46.7%)	4 (13.3%)^*^	1 (3.3%)^*^	0 (0.0%)^*^	0 (0.0%)^*^	33.974	<0.001
Hypotension^a^	5 (16.7%)	5 (16.7%)	6 (20.0%)	2 (6.7%)	6 (20.0%)	2.937	0.602
Desaturation^b^	1 (3.3%)	0 (0.0%)	1 (3.3%)	4 (13.3%)	3 (10.0%)	5.593	0.198
Body movement	13 (43.3%)	12 (40.0%)	8 (26.7%)	9 (30.0%)	8 (26.7%)	3.300	0.509
Coughing	8 (26.7%)	3 (10.0%)	9 (30.0%)	9 (30.0%)	7 (23.3%)	4.532	0.339
Dizziness	7 (23.3%)	5 (16.7%)	4 (13.3%)	4 (13.3%)	5 (16.7%)	1.440	0.837
Sore throat	0 (0%)	0 (0.0%)	0 (0.0%)	0 (0.0%)	1 (3.3%)	3.941	1.000
Hiccups	2 (6.7%)	1 (3.3%)	3 (10.0%)	5 (16.7%)	6 (20.0%)	5.705	0.222

^a^ Defined as systolic blood pressure <90 mmHg or <80% of baseline.

^b^ Defined as SpO_2_ < 95%.

* *P* < 0.005 compared with remimazolam 0 mg·kg-1, after Bonferroni correction.

## 4 Discussion

In this randomized, double-blind, up-and-down sequential dose-finding study, we investigated the dose–response relationship between remimazolam and propofol during sedated gastroscopy. The principal finding was a dose-dependent propofol-sparing effect of remimazolam supplementation. Among the tested doses, 0.1 mg·kg^-1^ emerged as the lowest dose associated with a clinically meaningful reduction in propofol requirement without prolonging recovery, and appeared to provide a favorable balance between sedative efficacy and recovery characteristics.

The favorable pharmacokinetic and pharmacodynamic properties of remimazolam, including rapid onset, high clearance, short context-sensitive half-time, and minimal tissue accumulation, support its suitability for procedural sedation (Kim, 2022). Previous studies have demonstrated that remimazolam provides rapid induction and recovery comparable to propofol, although delayed emergence may occur in the absence of flumazenil antagonism (Zhang et al., 2023). In addition, remimazolam has been associated with less injection pain and less cardiovascular depression than propofol. The propofol-sparing effect observed in our study likely reflects complementary pharmacodynamic mechanisms: remimazolam provides rapid γ-aminobutyric acid type A (GABAA) receptor–mediated hypnosis, while propofol titration via target-controlled infusion fine-tunes sedation depth. BIS monitoring helped standardize sedation depth across groups and supported protocolized titration. These findings are consistent with prior studies of remimazolam-propofol combinations (Li et al., 2024; Sun et al., 2024; Zhang et al., 2024). Our opioid-free protocol indicates that the observed propofol-sparing effect was primarily attributable to the interaction between these two hypnotic agents.

We demonstrated that remimazolam at doses of 0.1, 0.15, and 0.2 mg·kg^-1^ showed lower average Ceprop at loss of consciousness and lower calculated EC_50_ required for effective sedation, whereas 0.05 mg·kg^-1^ reduced EC_50_/Ceprop but did not significantly reduce propofol requirement. These findings suggest a dose-dependent propofol-sparing effect. Because remimazolam lacks intrinsic analgesic effects, the mechanism is likely related to enhanced depth of sedation that attenuates pharyngo-esophageal reflexes, thereby reducing airway–pharyngeal reflex responsiveness and stabilizing sedation conditions during insertion. Importantly, although higher doses (0.15 and 0.2 mg·kg^-1^) produced greater propofol sparing, they were also associated with delayed eye opening. In contrast, 0.1 mg·kg^-1^ achieved significant propofol reduction without prolonging emergence, supporting this dose as a practical adjunct to propofol for routine gastroscopy sedation.

Previous studies have reported that recovery after remimazolam may be prolonged at higher doses or without flumazenil reversal (Kamata and Masui, 2023; So et al., 2023; Yamamoto et al., 2021). In agreement with these findings, we observed that time to eye opening was significantly prolonged in the 0.15 and 0.2 mg·kg^-1^ groups compared with the control group. However, no differences in PACU duration were observed among the five groups, and recovery times were similar among the 0, 0.05, and 0.1 mg·kg^-1^ groups. These findings suggest that remimazolam at 0.1 mg·kg^-1^ may be safely incorporated into routine endoscopy workflows without compromising efficiency.

With respect to safety, remimazolam was associated with a significantly lower incidence of injection pain, while the incidences of hypotension, desaturation, body movement, coughing, dizziness, and sore throat were comparable among groups. Hiccups were observed in 17/150 patients (11.3%); however, the overall incidence did not differ significantly across the five dose groups (P = 0.222). Hiccups also occurred in the 0 mg·kg^-1^ group, suggesting a multifactorial etiology related to procedural stimulation and individual susceptibility. Hiccups have been noted as a potential adverse event during remimazolam-based procedural sedation and may merit clinical attention, although the underlying mechanism remains unclear (Huang et al., 2022). In our study, hiccups were generally transient and were managed conservatively, as no established specific therapy currently exists. No patient experienced bradycardia, postoperative nausea and vomiting, or intraoperative awareness. These results are consistent with prior pediatric and adult endoscopy studies and with recent meta-analytic evidence demonstrating that remimazolam reduces injection pain and cardiorespiratory adverse events compared with propofol (An et al., 2024; Chen et al., 2020). Collectively, our data did not identify a major safety signal under the conditions studied; however, safety findings should be interpreted as exploratory given the limited power for adverse-event comparisons.

Several strengths warrant mention. The study employed an opioid-free sedation protocol, allowing isolation of the sedative interaction between remimazolam and propofol without confounding analgesic effects. Target-controlled propofol infusion was used to minimize inter-operator variability in drug delivery, and propofol requirement was adjusted for body weight and anesthesia duration, improving the precision of dose comparisons.

This study also has limitations. First, Because propofol concentrations were derived from a Marsh-model TCI system rather than measured pharmacokinetic concentrations, the absolute Ce-based EC_50_ values may vary across different TCI implementations and alternative PK/PD models, such as Schnider or Eleveld. Therefore, these absolute values should be interpreted with caution, although within-study between-group comparisons remain valid because the same TCI device, targeting mode, and model framework were used throughout. In addition, because the Dixon up-and-down design generates sequential observations that are not fully independent, between-group comparisons of EC_50_ should be interpreted with caution. Second, several clinically relevant procedural sedation outcomes, including endoscopist satisfaction, patient satisfaction, sedation induction time, and the incidence or duration of BIS <40, were not prospectively included as predefined endpoints. Third, periprocedural outcomes were assessed only until PACU discharge, and longer-term recovery and delayed adverse events were not evaluated. In addition, the study was not specifically powered to detect moderate between-group differences in adverse-event rates, and therefore the safety findings should be interpreted as descriptive and exploratory. Fourth, the study was limited to gastroscopy, a relatively short procedure, and the extrapolation of these findings to longer or more stimulating procedures, such as colonoscopy or combined endoscopy, requires further investigation. Finally, pediatric, elderly, obese, and other higher-risk patients were excluded, limiting the generalizability of our findings. Future studies should focus on these higher-risk populations and explore the effects of remimazolam–propofol combinations on longer procedures, longer-term recovery, and broader sedation-quality and safety outcomes.

## 5 Conclusion

In summary, remimazolam at doses of 0.1–0.2 mg·kg^-1^ reduced the estimated propofol EC_50_ and propofol requirement for effective sedation during gastroscopy. Although time to eye opening was prolonged in the 0.15 and 0.2 mg·kg^-1^ groups compared with the 0 mg·kg^-1^ group (*P* < 0.005), no differences were observed among the 0, 0.05, and 0.1 mg·kg^-1^ groups (all *P* > 0.05), and PACU duration did not differ among groups. Taken together, these data suggest that remimazolam at 0.1 mg·kg^-1^ may provide a favorable balance between propofol-sparing efficacy and rapid recovery and may serve as a practical adjunct to propofol for sedated gastroscopy. Larger multicenter studies are warranted to confirm these findings and to further evaluate safety and recovery outcomes in higher-risk populations, which were excluded from this trial, particularly elderly and obese patients (BMI ≥30 kg·m^-2^).

## Data Availability

The data supporting the findings of this study are available from the corresponding authors upon reasonable request, subject to institutional and ethical requirements.
