# Supplementary material for: Dose optimization of remimazolam as an adjunct to propofol for gastroscopy sedation: a randomized double-blind trial
Source: Front Pharmacol. 2026 Apr 29;17:1782559. doi: 10.3389/fphar.2026.1782559 (PMC13168902; doi:10.3389/fphar.2026.1782559)
Supplement: Supplementary file 1 [file Table1.docx]

Supplementary Material

Supplementary Table S1 MOAA/S Scale

| **Score** | **Response to Stimulus** |
| --- | --- |
| 5 | Responds readily to name spoken in normal tone |
| 4 | Lethargic response to name spoken in normal tone |
| 3 | Responds only after name is called loudly and/or repeatedly |
| 2 | Responds only after mild prodding or shaking |
| 1 | Responds only to painful stimulus |
| 0 | No response to painful stimulus |

Supplementary Table S2 Bispectral Index Scale (BIS)

| BIS Value | Level of Sedation |
| --- | --- |
| > 80 | Inadequate sedation, imminent awakening |
| 40 - 60 | Appropriate anesthetic depth |
| < 40 | Excessive sedation, profound suppression |

Supplementary Table S3 Visual Analog Scale (VAS) Score

| **Score** | **Pain Level** |
| --- | --- |
| 0 | No Pain |
| 1 - 3 | Mild Pain, tolerable |
| 4 - 6 | Moderate Pain, interferes with sleep, still tolerable |
| 7 - 10 | Severe Pain, difficult to tolerate |

Supplementary Table S4 Comparison of MAP, HR, and SpO₂ among 5 groups of patients at different time points

| **Group** | **Time** | **MAP (mmHg)** | **HR (beats/min)** | **SpO₂ (%)** |
| --- | --- | --- | --- | --- |
| Remimazolam 0 mg·kg⁻¹ | T1 | 103.17±9.65 | 86.27±18.92^b^ | 99 (98, 100)^c^ |
|  | T2 | 89.20±10.47^ab^ | 83.67±10.79^ab^ | 100 (99, 100)^c^ |
|  | T3 | 89.77±12.34^ab^ | 81.23±11.03^ab^ | 99 (98, 100) |
|  | T4 | 83.67±12.12^a^ | 75.97±10.27 | 99 (98, 99) |
| Remimazolam 0.05 mg·kg⁻¹ | T1 | 97.93±9.81 | 77.53±12.54^b^ | 99 (99, 100)^c^ |
|  | T2 | 86.63±11.70^ab^ | 82.20±11.19^ab^ | 100 (99, 100)^c^ |
|  | T3 | 87.27±11.41^ab^ | 77.87±11.87^ab^ | 99 (98, 100) |
|  | T4 | 84.33±10.22^a^ | 70.17±9.24 | 98 (97, 99) |
| Remimazolam 0.1 mg·kg⁻¹ | T1 | 98.90±11.93 | 77.40±16.81^b^ | 99 (98, 100)^c^ |
|  | T2 | 88.29±12.92^ab^ | 84.17±12.29^ab^ | 99 (98, 100)^c^ |
|  | T3 | 88.63±13.97^ab^ | 87.30±12.98^ab^ | 99 (98, 100) |
|  | T4 | 84.50±11.98^a^ | 74.37±14.30 | 98 (98, 99) |
| Remimazolam 0.15 mg·kg⁻¹ | T1 | 98.50±12.69 | 79.53±12.60 | 99 (98, 100)^c^ |
|  | T2 | 88.20±12.48^a^ | 86.93±14.30^a^ | 99 (98, 100)^c^ |
|  | T3 | 88.83±13.66^a^ | 87.33±12.94^a^ | 99 (98, 100) |
|  | T4 | 84.77±11.02^a^ | 77.47±11.09 | 99 (98, 99) |
| Remimazolam 0.2 mg·kg⁻¹ | T1 | 99.93±11.94 | 78.27±10.41 | 100 (99, 100)^c^ |
|  | T2 | 86.80±10.81^a^ | 83.27±8.63^a^ | 100 (98, 100)^c^ |
|  | T3 | 85.10±11.17^a^ | 84.87±10.42^a^ | 99 (98, 100) |
|  | T4 | 82.97±10.45^a^ | 72.80±9.42 | 99 (98, 99) |

Data are presented as mean ± SD or median (IQR), as appropriate.

^a^ *P* < 0.005 compared with T1 within the same group after Bonferroni correction.

^b^ *P* < 0.005 compared with T4 within the same group after Bonferroni correction.

^c^ *P* < 0.005 compared with T3 and T4 within the same group after Bonferroni correction.
