# Supplementary figures and images for: Dose optimization of remimazolam as an adjunct to propofol for gastroscopy sedation: a randomized double-blind trial
Source: Front Pharmacol. 2026 Apr 29;17:1782559. doi: 10.3389/fphar.2026.1782559 (PMC13168902; doi:10.3389/fphar.2026.1782559)

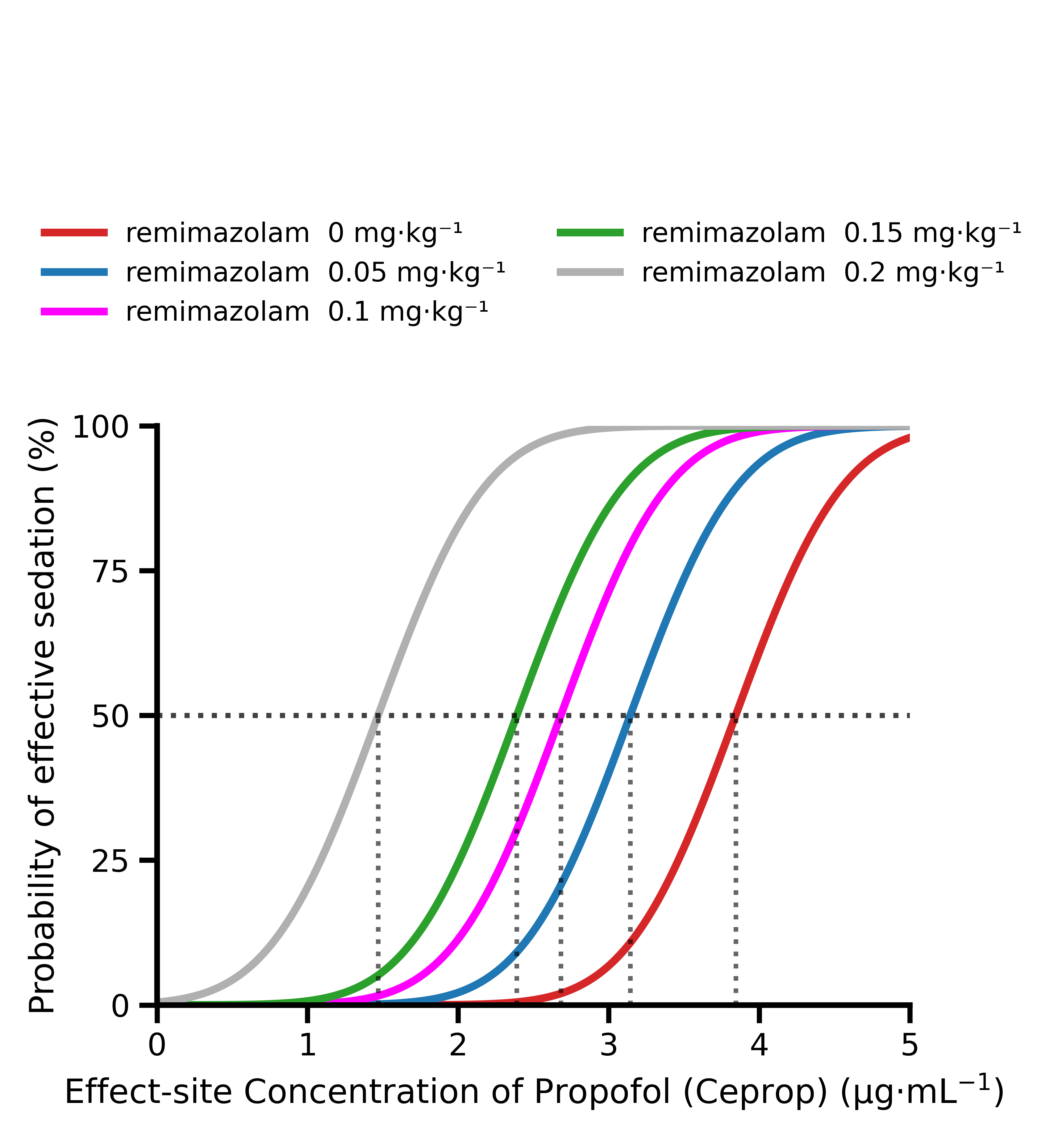

Supplement: Supplementary file 2 [file Image1.png]
